# Blue Light Receptor PHR2‐Mediated Spectrum Antiviral Immunity

**DOI:** 10.1002/advs.78050

**Published:** 2026-09-30

**Authors:** Runyi Chen, Yuxin Tai, Xinyu Wang, Chunhua Mu, Xia Liu, Gongjian Li, Jiayi Liu, Xin Lu, Duo Shi, Zhiqiang Zhou, Jienan Han, Zhuanfang Hao, Mingshun Li, Degui Zhang, Hongjun Yong, Zhennan Xu, Jianfeng Weng, Xinhai Li

**Affiliations:** ^1^ State Key Laboratory of Crop Gene Resources and Breeding Institute of Crop Sciences Chinese Academy of Agricultural Sciences Beijing China; ^2^ National Key Laboratory of Smart Farm Technology and System College of Agriculture Northeast Agricultural University Harbin China; ^3^ Shandong Academy of Agricultural Sciences Jinan China; ^4^ Hinggan League Academy of Agricultural and Animal Husbandry Sciences Hinggan League China; ^5^ National Nanfan Research Institute (Sanya) Chinese Academy of Agricultural Sciences Sanya China

**Keywords:** blue light, ISP, PHR2, reactive oxygen species, rice black streaked dwarf virus

## Abstract

Plant viruses have evolved sophisticated virulence strategies to counteract host antiviral defenses. Blue light, as an important environmental signal, can regulate plant growth and tolerance to biological stress. Nevertheless, how blue light regulates plant responses to antiviral immunity remains largely obscure. Here, we demonstrate that blue light suppresses rice black‐streaked dwarf virus (RBSDV) replication. The blue light receptor ZmPHR2 functions as a key immune component, positively regulating maize resistance to RBSDV. Mechanistically, ZmPHR2 interacts with ZmISP in chloroplasts to trigger a defensive reactive oxygen species (ROS) burst, thereby conferring resistance. The RBSDV effector P6 enhances the ZmPHR2‐ZmISP interaction, alters their subcellular distribution, and suppresses ROS accumulation. A functionally analogous P6‐OsPHR2‐OsISP module similarly mediates RBSDV resistance in rice. Our findings reveal that blue light perception directly activates antiviral defense pathways, uncovering a molecular basis for RBSDV resistance. This study provides a theoretical basis for artificially modifying the blue light receptor protein to enhance the resistance of cereal crops.

## Graphical abstract

Blue light suppresses RBSDV through the blue‐light receptor ZmPHR2, which interacts with ZmISP in chloroplasts to trigger ROS burst and antiviral defense. The viral effector P6 enhances ZmPHR2–ZmISP interaction, alters their distribution, and suppresses ROS. This mechanism is conserved in rice, providing a target for enhancing crop resistance.

## 1 Introduction

The outcome of plant‐pathogen interactions is influenced by multiple factors, including environmental conditions [1], host defense mechanisms [2], and the pathogen's ability to evade or overcome these defenses [3]. Light, as one of these environmental variables, serves not only as the primary energy source for photosynthesis but also as a key environmental signal that modulates immunity in both plants and animals [4]. For instance, low‐dose blue light exposure on the skin has been shown to activate innate immune responses without causing damage, and can even suppress tumor growth and metastasis [5]. Throughout evolution, plants have developed a suite of photoreceptors to perceive dynamic changes in light quality and intensity. Among these, cryptochromes (CRYs) function as blue light receptors that mediate a variety of blue light‐dependent responses across both plant and animal kingdoms. CRYs are highly conserved, present in organisms ranging from bacteria to higher eukaryotes [6, 7]. In animals, they constitute core components of the circadian clock. Furthermore, in migratory species such as monarch butterflies and birds, CRYs play a role in sensing the Earth's magnetic field, thereby facilitating navigation during long‐distance migration [8]. In plants, cryptochrome (CRY) mainly act as blue light receptors, mediating various photobiological responses [9]. *Cryptochrome 1* (*CRY1*) is the first blue light receptors identified in *Arabidopsis thaliana* [10] and involved photomorphogenesis [11], photoperiodic flowering [12], circadian rhythm [13], stomatal opening and closing [14], shade avoidance response [15], tropic growth [16], root elongation [17] and non‐biological stress response [18]. Recent studies in crops have shown that CRYs are involved in regulating a variety of important agronomic traits, including the germination of barley seeds [19], the stress response of wheat [20], the plant architecture of soybeans, and the flowering period of rice [7, 21]. Furthermore, blue light and its receptors enhance pattern‐triggered immunity (PTI) against microbial pathogens [14]. For instance, the potato photoreceptors *Stphot1* and *Stphot2* play a role as susceptibility factors in the interaction between plants and pathogens [22]. Both promote the infection process of the pathogenic phytophthora on plants in a non‐redundant manner. Currently, the functions of blue light and its photoreceptors in plant growth and development are well characterized, their roles in antiviral immunity and other defense processes remain largely unexplored.

Plant viruses, predominantly transmitted by biotic vectors, pose a severe threat to global security through extensive crop damage [23]. To establish a systemic infection, the viruses must hijack host cellular mechanisms for replication and movement. Remarkably, the effectiveness of viral stems largely from the sophisticated virulence strategies mediated by viral effector proteins, which are adept at disrupting plant immune responses [24]. A prominent example is the RNA silencing suppressor, virally encoded proteins that specifically inhibit the host's post‐transcriptional gene silencing (PTGS) machinery, a major layer of antiviral defense [25, 26]. Despite their critical role in infection, the precise modes of action and functions of RNA silencing suppressors remain elusive. Thus, a deeper understanding of these effector proteins is crucial, not only to advance fundamental knowledge of plant‐virus interactions but also to inform the development of novel genetic and chemical strategies for durable resistance.

A wide range of RNA viruses are capable of infecting maize, posing serious threats to its yield and quality worldwide [27]. For example, rice black‐streaked dwarf virus (RBSDV), a type of virus caused maize rough dwarf disease (MRDD), is a globally devasting viral disease transmitted by the small brown planthopper (*Laodelphax striatellus*) and referred to as the “cancer” of maize [28, 29]. MRDD typically induces severe growth abnormalities, including plant dwarfism, internode shortening, abnormal ears and tassels [30]. The genome of RBSDV consists of ten double‐strand RNA (dsRNA) segments (*S1*‐*S10*), encoding thirteen proteins, including the effector proteins P6 and P8 [31]. Previous studies have shown that the independently evolved and functionally conserved viral transcriptional repressor RBSDV‐P8 employs divergent molecular strategies to target and suppress the central component of the auxin signaling pathway *OsARF17* [32]. Concurrently, P8 proteins specifically interact with key jasmonic acid (JA) signaling transcription factors OsMYC2 and OsMYC3, resulting in the coordinated suppression of both auxin‐ and JA‐mediated antiviral responses [33]. This dual targeting strategy effectively disrupts the host's defense network and creates a favorable cellular environment for viral replication and pathogenesis. The RNA silencing suppressor protein P6 encoded by RBSDV enhances the ubiquitination modification of small ubiquitin‐like modifiers (SUMO) conjugating enzyme 1b (*OsSCE1b*) and promotes its degradation, thereby inhibiting the SUMOylation modification of *OsPELOTA* and the rice antiviral RNA decay defense response [34]. Although viral‐encoded RNA silencing suppressors are well‐established key virulence factors that counteract plant immunity, their potential involvement in other pathways and possible synergistic effects on viral infection are an important and unresolved question.

Plants have evolved diverse defense strategies to combat RBSDV, including the disease‐resistant protein ZmGDIα‐hel, which reduces the active gibberellin levels induced by viral infection [35, 36], and the ZmDBF2‐ZmGLK36‐ZmJMT/ZmLOX8 molecular module, which coordinates transcriptional reprogramming to enhance antiviral immunity [37]. Notably, emerging evidence underscores the pivotal role of light signaling in regulating viral immune responses, revealing a previously unexplored layer of plant immunity. For example, near‐infrared light irradiation promotes the accumulation of PIF4 in plants, a phytochrome‐interacting factor, which in turn upregulates the transcription of *RDR6* and *AGO1*, thereby strengthening the RNA interference (RNAi)‐mediated antiviral pathway [38]. However, viruses have evolved countermeasures. For instance, viruses target NbPIF4 by encoding the βC1 protein, suppressing its transcriptional activity, and thereby antagonizing the RNAi antiviral effect mediated by PIF4 [39]. Beyond near‐infrared signaling, blue light, a key component of the visible spectrum, is well known to regulate plant growth [17], development [40], and stress responses [14]. Nevertheless, whether blue light and its corresponding receptors participate in plant resistance to RBSDV and the molecular mechanisms that may underpin such regulation remain entirely unknown.

In this study, we show that blue light represses viral replication and identify the blue light receptor *ZmPHR2* as a positive regulator of resistance against RBSDV. ZmPHR2 interacts with ZmISP, and the resulting complex localizes to chloroplasts, where it enhances ZmISP reactive oxygen species (ROS) production. Furthermore, the viral effector RBSDV P6 disrupts the ZmPHR2‐ZmISP complex, redirecting its localization from chloroplasts to the cytoplasm and thereby suppressing ROS accumulation. This regulatory mechanism of P6‐OsPHR2‐OsISP is conserved in rice, suggesting its potential to confer resistance in cereal crops and providing a strategy for breeding RBSDV‐resistant varieties.

## 2 Results

### 2.1 Blue Light Inhibits RBSDV Replication Through PTI

Blue light serves as a key environmental signal regulating plant growth and development, playing a critical role in balancing photosynthesis and defense responses in dynamic conditions [17]. To investigate whether blue light also participates in antiviral defense, a photoperiod‐controlled experiment was designed to dissect blue light's antiviral function: Maize plants were grown under either blue LEDs (λ_p__e__a__k_ = 480 nm, 31 µmol m^−2^s^−1^) or white light controls (34 µmol m^−2^s^−1^) (Figure 1A). Inoculation assays confirmed that blue light treatment significantly suppressed RBSDV accumulation, as indicated by markedly reduced expression levels of RBSDV *S6*, *S8*, and *S10* compared to plants grown under white light (Figure 1B–D). Compared to white light, blue light treatment accumulated much higher levels of ROS (Figure S1A–C), indicating constitutive defense activation. The early PTI responses include the acidification of the periclinal space, the production of ROS, and the reprogramming of transcription [41]. The later responses involve the biosynthesis of defense‐related plant hormones, such as salicylic acid (SA) and jasmonic acid (JA) [42, 43]. RT‐qPCR analysis revealed that marker genes *ZmNPR1*, *ZmEDS1*, *ZmAOC*, and *ZmAOS* from the SA and JA pathways were significantly upregulated after blue light treatment (Figure S1D–G), supporting the engagement of later defense responses, including hormone‐mediated immunity. These data suggest a link between ROS metabolism, SA, JA, and RBSDV in blue light‐mediated resistance to RBSDV.

**FIGURE 1:**
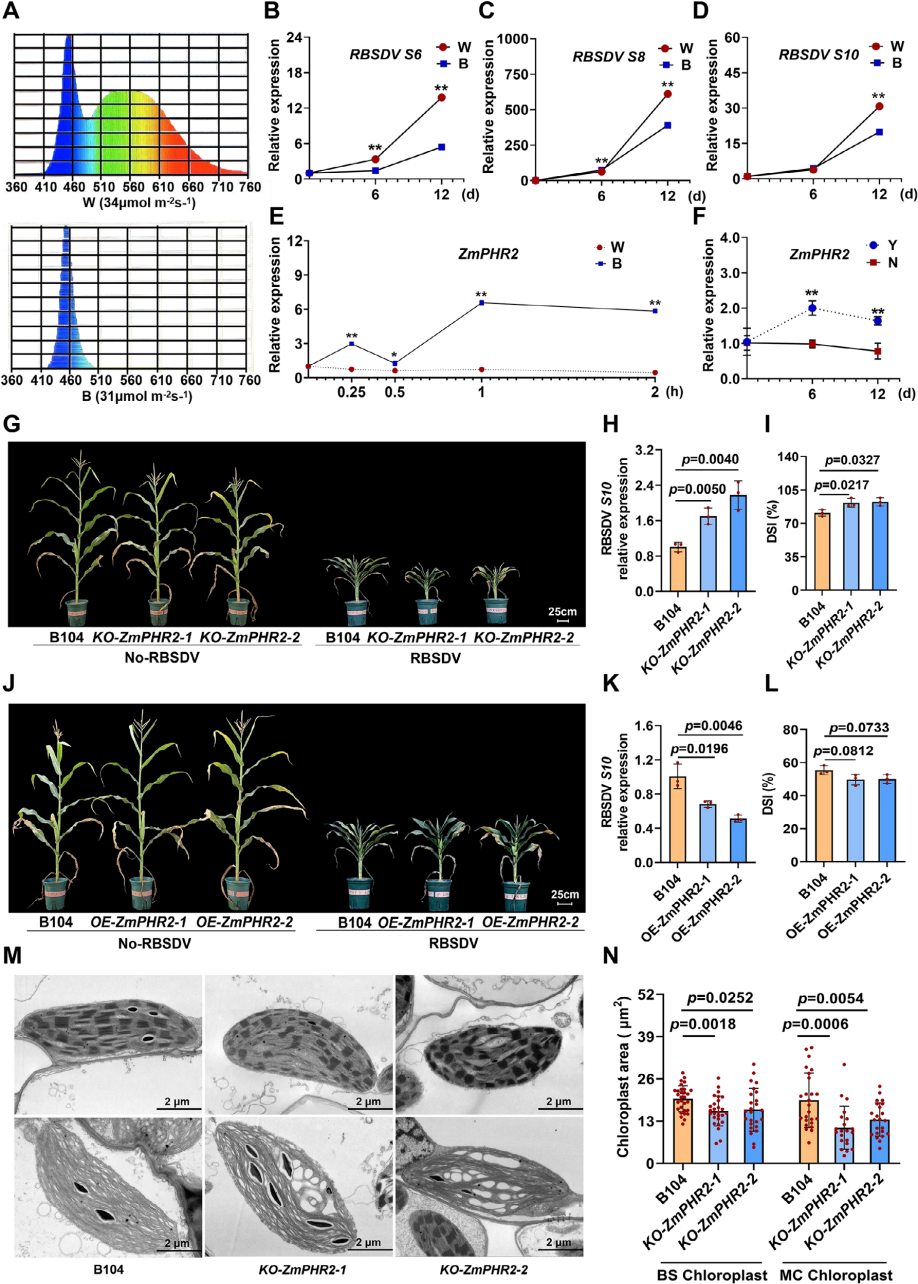
Blue light‐mediated *ZmPHR2* positively regulates maize resistance to RBSDV. (A) Light spectral composition of white light (W) and blue light (B) used in experiments. (B–D) Blue light inhibits the accumulation of the effector protein of RBSDV. The expression levels of RBSDV *S6* (B), RBSDV *S8* (C), and RBSDV *S10* (D) were detected in inoculated B73 plants treated with blue light (B, marked with blue dots) or white light (W, marked with red dots) at 6 days and 12 days during the V3 stage. Data are means ± SD (*n* = 5 biologically independent samples with six plants per biological replicate). (E,F) *ZmPHR2* was significantly induced at all time points after blue light treatment and RBSDV inoculation, respectively. Relative expression level of *ZmPHR2* in response to blue light and white light treatment (E) and RBSDV inoculation (F) (Y: RBSDV‐inoculated plants, represented by blue dots; N, no‐inoculated with RBSDV, represented by red dots). Data are means ± SD (*n* = 3 biologically independent samples with six plants per biological replicate). (G) MRDD phenotypes of B104 and *ZmPHR2* knockout plants under indicated treatments. No‐RBSDV, plants not inoculated with RBSDV; RBSDV, plants were artificially inoculated with RBSDV at the V3 stage. Scale bar = 25 cm. (H) Relative expression level of RBSDV *S10* in B104 and *ZmPHR2* knockout plants at 36 days post‐inoculation. Data are means ± SD (*n* = 3 biologically independent samples with six plants per biological replicate). (I) DSI values of B104 and *ZmPHR2* knockout plants. Data are means ± SD from 3 biological replicates (*n* = 20 plants per replicate). (J) MRDD phenotypes of B104 and *ZmPHR2* overexpression plants under indicated treatments. Scale bar = 25 cm. (K) The relative expression level of RBSDV *S10* in B104 and *ZmPHR2* overexpression plants. Data are means ± SD (*n* = 3 biologically independent samples with six plants per biological replicate). (L) DSI values of B104 and *ZmPHR2* overexpression plants. Data are means ± SD (*n* = 3 biologically independent samples with 20 plants per replicate). (M, N) Morphological changes in chloroplast morphology in BS and MC cells. For BS cells: Chloroplast width and height were measured in B104 (*n* = 30), *KO‐ZmPHR2‐1* (*n* = 27) and *KO‐ZmPHR2‐2* (*n* = 25) plants. For MC cells: Chloroplast width and height were analyzed in B104 (*n* = 25), *KO‐ZmPHR2‐1* (*n* = 21) and *KO‐ZmPHR2‐2* (*n* = 23) plants. BS: bundle sheath; MC: mesophyll cell. Statistical significance was determined using Students’ *t*‐test. ^*^ *p *< 0.05, ^**^ *p* < 0.01 are used to label significant differences in (B–F); no labels are shown for comparisons with *p* > 0.05. Exact *p* values are directly marked on the (H, I, K, L and N).

### 2.2 *ZmPHR2* Positively Regulate Defense Against RBSDV

To identify the photoreceptor mediating blue light‐triggered antiviral immunity, nine *ZmCRY* genes were identified based on domain analysis (Figure S2A). Under blue light, *ZmCRY1a1*, *ZmCRY3*, and *ZmPHR2* were continuously upregulated by RT‐qPCR (Figures 1E and S2B–I). Following RBSDV infection, only *ZmPHR2* was significantly induced at both 6 and 12 dpi, respectively (Figures 1F and S3A–H). These results suggest that *ZmPHR2* acts as a key regulator in blue light‐dependent antiviral responses, mediating the suppression of RBSDV replication.

To functionally validate *ZmPHR2*’s role in blue light‐mediated antiviral immunity, we performed systematic characterization of its expression pattern and conducted genetic and phenotypic analysis. RT‐qPCR showed that *ZmPHR2* was highly expressed in leaves, with protein levels increased by both blue light and RBSDV infection (Figure S4A–C). We obtained *zmphr2* mutants (EMS‐induced stop‐gain) in B73 background and two independent *ZmPHR2* knockout lines (*KO*‐*ZmPHR2*‐1 and *KO*‐*ZmPHR2*‐2) in B104 background (Figure S5A,B). Inoculation assay showed that the loss‐of‐function mutants (EMS‐induced and CRISPR/Cas9 knockouts) exhibited higher virus RNA levels (*S6*, *S8*, *S10*) and more severe disease symptoms in RBSDV‐infected mutants than in B73 or B104, along with developmental defects such as reduced plant height, ear height and chlorophyll content (SPAD value) (Figures 1G–I and S5C–O). Conversely, two overexpression lines (*OE*‐*ZmPHR2*‐1 and *OE‐ZmPHR2*‐2) exhibited enhanced resistance, with lower RBSDV *S6*, *S8* and *S10* expression levels and reduced disease severity index (DSI) compared with B104 (Figures 1J–L and S6A–D). The overexpression lines also showed a significant increase in plant height and relative chlorophyll content (Figure S6E–G). These results demonstrate that *ZmPHR2* positively regulates resistance to RBSDV. Since blue light signals mainly function in chloroplasts, we examined chloroplast structures in transgenic materials by transmission electron microscopy (TEM). These observations revealed that *ZmPHR2* knockout lines disrupted chloroplast integrity, with malformed and smaller chloroplasts in both mesophyll cells (MC) and bundle sheath cells (BS) (Figures 1M,N and S6H,I). Collectively, these results demonstrate that *ZmPHR2* functions as a key positive regulator in blue light‐mediated antiviral immunity, while also contributing to normal plant growth and development.

### 2.3 ZmPHR2 Recruits ZmISP to Potentiate Antiviral Defense to RBSDV

To identify the biological roles of the *ZmPHR2*, we initially used ZmPHR2 as bait to screen a maize complementary DNA (cDNA) library by yeast two‐hybrid (Y2H) assay (Table S2). Sequence analysis revealed that GRMZM2G162748, which encodes the iron‐sulfur cluster subunit protein ZmISP, serves as a potential interacting partner of ZmPHR2. The amino acid sequence of ZmISP shares 82.38% homology with TaISP from *Triticum aestivum* [44]. Similar to TaISP, ZmISP is a nuclear gene encoded chloroplast and possesses a typical chloroplast transit peptide (cTP), as well as CytB6‐F_Fe‐S and Rieske domains (Figure S7A,B). To validate the interaction between ZmPHR2 and ZmISP, the full‐length sequence of ZmPHR2 fused with the GAL4 DNA‐binding domain (BD) and that of ZmISP fused with the GAL4 activation domain (AD) were co‐expressed in yeast cells. As expected, the Y2H results showed that ZmPHR2 specifically interacted with ZmISP (Figure 2A). The interaction was further confirmed by in *vitro* pull‐down assays and luciferase complementation imaging (LCI) (Figure 2B,C). Next, bimolecular fluorescence complementation (BiFC) assays were then used to test whether the interaction also occurred in vivo. Strong fluorescence signals were detected in chloroplasts of protoplasts transiently co‐expressing ZmISP‐nYFP and ZmPHR2‐cYFP (Figure 2D), but not in the negative controls. Together, these results demonstrate that ZmPHR2 physically interacts with ZmISP.

**FIGURE 2:**
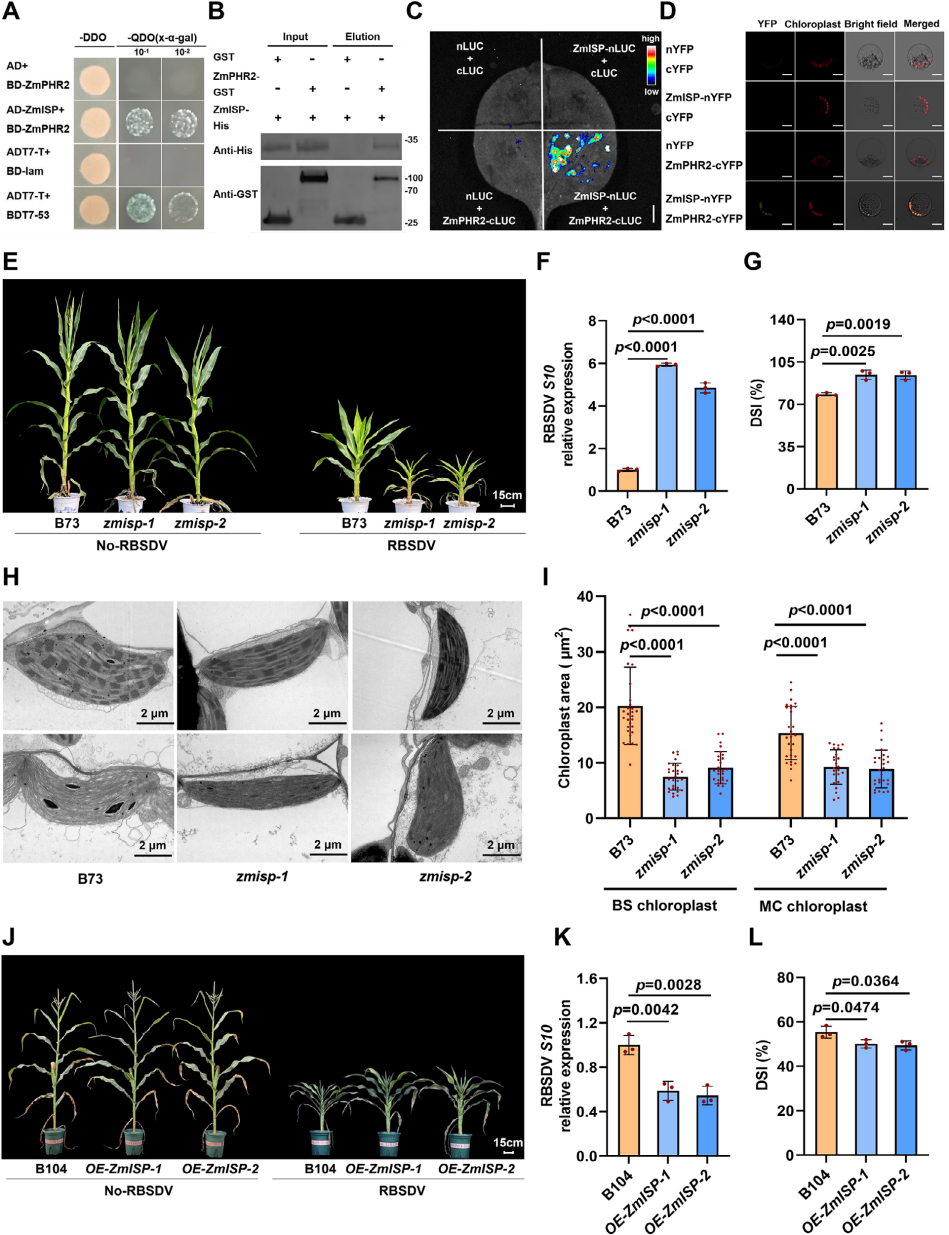
*ZmISP* positively regulates maize resistance to RBSDV. (A) Y2H assay showing the interaction between ZmPHR2 and ZmISP. (B) Pull‐down assay shows that ZmPHR2 directly interacts with ZmISP in *vitro*. (C) LCI assays in *N.benthamiana* leaves showing the interaction between ZmPHR2 and ZmISP. Scale bar = 1 cm. (D) BiFC assay shows the interaction between ZmPHR2 and ZmISP in maize protoplast cells. The interaction between cYFP and ZmISP‐nYFP or nYFP and ZmPHR2‐cYFP serves as the negative control. Scale bar = 10 µm. (E) MRDD phenotypes of B73 and *zmisp* mutants under indicated treatments. Scale bar = 15 cm. (F) Relative expression level of RBSDV *S10* in B73 and *zmisp* mutants after RBSDV inoculation. Data are means ± SD (*n* = 3 biologically independent samples with six plants per biological replicate). (G) DSI values of B73 and *zmisp* mutants after RBSDV inoculation. Data are means ± SD from 3 biological replicates (*n* = 20 plants per replicate). (H, I) Morphological changes in chloroplast morphology in BS and MC cells. For BS cells: Chloroplast dimensions were measured in B73 (*n* = 30), *zmisp‐1* (*n* = 28) and *zmisp‐2* (*n* = 27) plants. For MC cells: Chloroplast width and height were analyzed in B73 (*n* = 29), *zmisp‐1* (*n* = 24) and *zmisp‐2* (*n* = 27) plants. Scale bar = 2 µm. (J) MRDD phenotypes of B104 and *ZmISP* overexpression plants under indicated treatments. Scale bar = 15 cm. (K) The relative expression level of RBSDV *S10* in B104 and *ZmISP* overexpression plants after RBSDV inoculation. Data are means ± SD (*n* = 3 biologically independent samples with six plants per biological replicate). (L) DSI values of B104 and *ZmISP* overexpression lines after RBSDV inoculation. Data are means ± SD from 3 biological replicates (*n* = 20 plants per replicate). Statistical significance was determined using Students’ *t*‐test. Exact *p* values are directly marked on the (F, G, I, K and L).

*ZmISP* exhibits a leaf‐predominant expression pattern, consistent with that of *ZmPHR2* and with its role in blue light and RBSDV infection (Figure S7C–E). We next asked whether ZmISP, as an interacting partner of ZmPHR2, contributes to antiviral immunity against RBSDV. Two homozygous *zmisp* mutants were obtained in B73 background (Figure S8A). Inoculation assay showed that the *zmisp* mutants displayed increased virus RNA levels (*S6*, *S8*, *S10*) and DSI (Figures 2E–G and S8B,C), along with developmental defects and abnormal chloroplast ultrastructure characterized by internal cavities and reduced size (Figures 2H,I and S8D,E). The *zmisp* mutants showed significant reductions in plant height, ear height and relative chlorophyll content (Figure S8F–H). Conversely, two overexpression lines (*OE*‐*ZmISP*‐1 and *OE*‐*ZmISP*‐2) displayed enhanced resistance and reduced virus RNA levels (*S6*, *S8*, *S10*) relative to B104 (Figures 2J–L and S9A–C). The overexpression lines also showed a significant increase in plant height and relative chlorophyll content (Figure S9D–F). These results demonstrate that *ZmISP* functions in coordination with *ZmPHR2* within a shared pathway to positively regulate antiviral defense and normal growth in maize.

### 2.4 ZmPHR2 Promotes ZmISP‐Mediated ROS Production in Chloroplasts

ZmISP is a component of the chloroplast Cyt b6/f complex. Although TaISP has a demonstrated role in regulating ROS metabolism in wheat against stripe rust [44], it remains unknown whether ZmISP performs an analogous function in the ROS pathway during maize infection by RBSDV. To investigate and elucidate whether the resistance mechanism mediated by the ZmPHR2‐ZmISP complex is involved in ROS metabolism, we conducted transcriptome analysis based on B104 and overexpression lines of *ZmPHR2* and *ZmISP*. 1702 and 1956 differentially expressed genes (DEGs) were identified in *ZmPHR2* and *ZmISP* overexpression lines, respectively (Tables S3 and S4). Kyoto Encyclopedia of Genes and Genomes (KEGG) enrichment analysis identified multiple ROS‐related pathways among the differentially expressed genes. Phenylpropanoid and flavonoid biosynthesis, both of which are linked to antioxidant production [45, 46], were enriched in both *OE*‐*ZmPHR2* and *OE*‐*ZmISP*. Whereas glutathione metabolism, the peroxisome pathway, and carotenoid biosynthesis, which are associated with ROS scavenging, H_2_O_2_ metabolism, and photoprotection, respectively [47, 48, 49, 50], were specifically enriched in *OE*‐*ZmISP* (Figures 3A,B and S10A,B; Tables S5 and S6). These results suggest that the ZmPHR2‐ZmISP module suppresses viral replication by orchestrating ROS‐mediated defense signaling.

**FIGURE 3:**
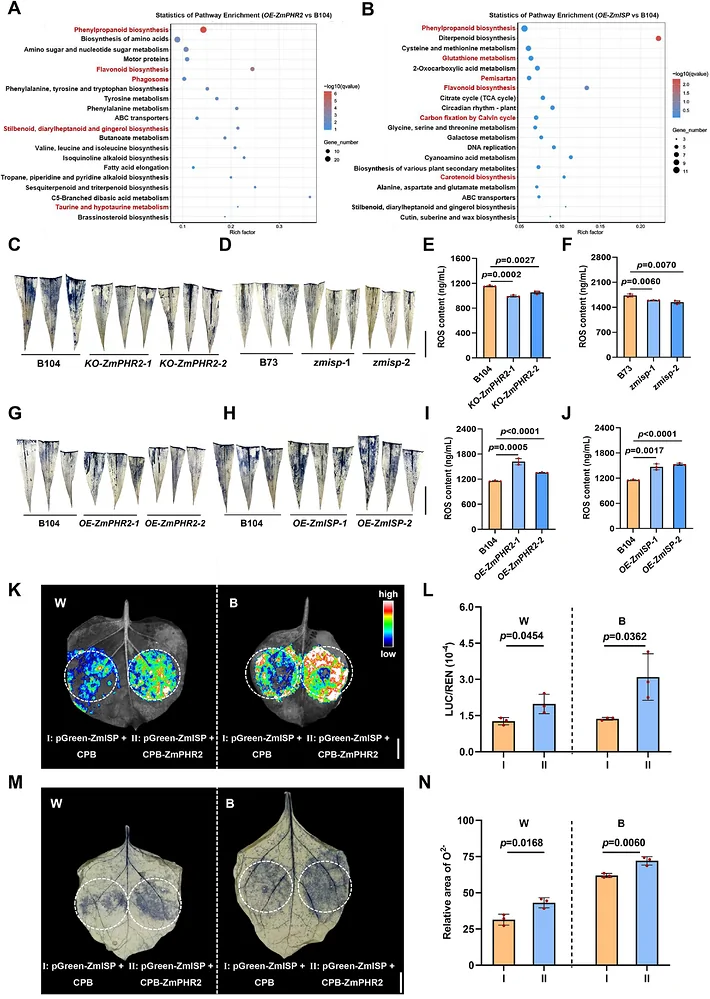
*ZmPHR2* and *ZmISP* promote the production of reactive oxygen species. (A, B) KEGG analysis of DEGs in B104, *ZmPHR2* (A) and *ZmISP* (B) overexpression plants. Red characters represent ROS‐related pathways. (C–F) NBT staining and relative ROS content of inoculated B104, B73, *ZmPHR2* knockout and *zmisp* mutants. Scale bar = 5 cm. Data are means ± SD (*n* = 3 biologically independent samples). (G–J) NBT staining and relative ROS content of inoculated B104, *ZmPHR2* and *ZmISP* overexpression plants after RBSDV inoculation. Data are means ± SD (*n* = 3 biologically independent samples). (K–L) Transient expression assay in *Nicotiana benthamiana* leaves showing ZmISP‐LUC activity upon co‐expression with ZmPHR2 or the empty vector under white (W) and blue (B) light. pGreen‐ZmISP and *CPB* were used as negative controls. Data are means ± SD (*n* = 3 biologically independent samples). Scale bar = 1 cm. (M, N) NBT staining for ROS detection and relative area in *Nicotiana benthamiana* leaves. W: white light; B: blue light. Data are means ± SD (*n* = 3 biologically independent samples). Statistical significance was determined using Students’ *t*‐test. Exact *p* values are directly marked on the (E, F, I, J, L and N).

To assess the effect of RBSDV infection on the regulation of ROS production by *ZmPHR2* and *ZmISP*, we measured ROS levels via nitro blue tetrazolium (NBT) staining and enzyme‐linked immunosorbent assay (ELISA). Both *ZmPHR2* and *ZmISP* mutants exhibited significantly reduced ROS accumulation and diminished leaf staining after viral challenge (Figures 3C–F and S10C,D), whereas overexpression lines of either gene showed enhanced ROS production and more extensive staining (Figures 3G–J and S10E,F). Consistent with this, under non‐infection conditions, overexpression lines displayed enhanced ROS accumulation and expanded staining areas relative to WT, whereas the mutants consistently showed the opposite effect (Figure S10G–K). These results confirm that ZmPHR2 and ZmISP positively regulate ROS accumulation through reactive oxygen metabolic pathways, thereby contributing to maize resistance against RBSDV.

To determine whether ROS production is required for the antiviral function of *ZmPHR2* and *ZmISP*, B104, *OE‐ZmPHR2* and *OE‐ZmISP* plants were pretreated with the ROS scavenger N‐acetyl‐L‐cysteine (NAC) [51] prior to RBSDV inoculation. The effective scavenging of ROS by NAC was verified by NBT staining, which showed 40 mM NAC significantly reduced ROS content (Figure S11A,B). NAC application led to significantly higher transcript levels of RBSDV *S6* and *S8* in *OE*‐*ZmPHR2* and *OE*‐*ZmISP*, compared with water‐sprayed controls (Figure S11C, D). Notably, transcript levels of *S10* in NAC‐treated overexpression lines showed no statistical difference from those in B104 (Figure S11E), suggesting that resistance against *S10* was fully abolished when ROS was depleted. Collectively, these results confirm that *ZmPHR2* promotes *ZmISP*‐mediated ROS production, and the ROS accumulation is indispensable for antiviral defense against RBSDV.

Since *ZmPHR2* and *ZmISP* are required for ROS accumulation and resistance, to determine whether ZmPHR2 and ZmISP function cooperatively in a light‐dependent manner, we performed dual‐luciferase assays and transient expression in *Nicotiana benthamiana*. Luciferase activity was significantly enhanced upon co‐expression of ZmPHR2 and ZmISP, with a pronounced increase under blue light compared to white light (Figure 3K,L). Consistent with this, co‐expression of both genes in *Nicotiana benthamiana* leaves induced stronger ROS accumulation and larger NBT staining areas than expression of either gene alone, with the strongest effect observed under blue light (Figure 3M,N). These findings indicate that ZmPHR2 and ZmISP synergistically enhance ROS production, and their cooperative activity is potentiated by blue light.

### 2.5 RBSDV P6 Alters Subcellular Localization of the Antiviral ZmPHR2‐ZmISP Complex

Given the established role of the ZmPHR2‐ZmISP module in resistance to RBSDV (Figure 2A–D). We sought to determine how RBSDV interfaces with this blue light‐mediated pathway. To this end, we screened 13 viral proteins encoded by RBSDV using Y2H assay and found that ZmPHR2 specifically interacts with RBSDV P6 (Figure S12A). We further confirmed this interaction through LCI and pull‐down assays, while BiFC assays indicated that it occurs primarily in the cytoplasm (Figure 4A–C). To investigate the virulence function of P6, we generated overexpression lines of P6 (*OE*‐*P6*‐1 and *OE‐P6*‐2). Following artificial inoculation with RBSDV, the overexpression lines exhibited significantly increased RNA expression levels of *S6, S8*, and *S10*, along with a significant increase DSI (Figures 4D–G and S12B). The overexpression lines also exhibited typical infection symptoms, including reduced plant height and ear height, as well as lower relative chlorophyll content (Figure S12C–E). Notably, ROS accumulation was significantly suppressed in overexpression lines (Figures 4H–J and S12F–H). Together, these findings demonstrate that RBSDV P6 acts as a key virulence effector that facilitates viral infection by attenuating ROS‐mediated defense responses.

**FIGURE 4:**
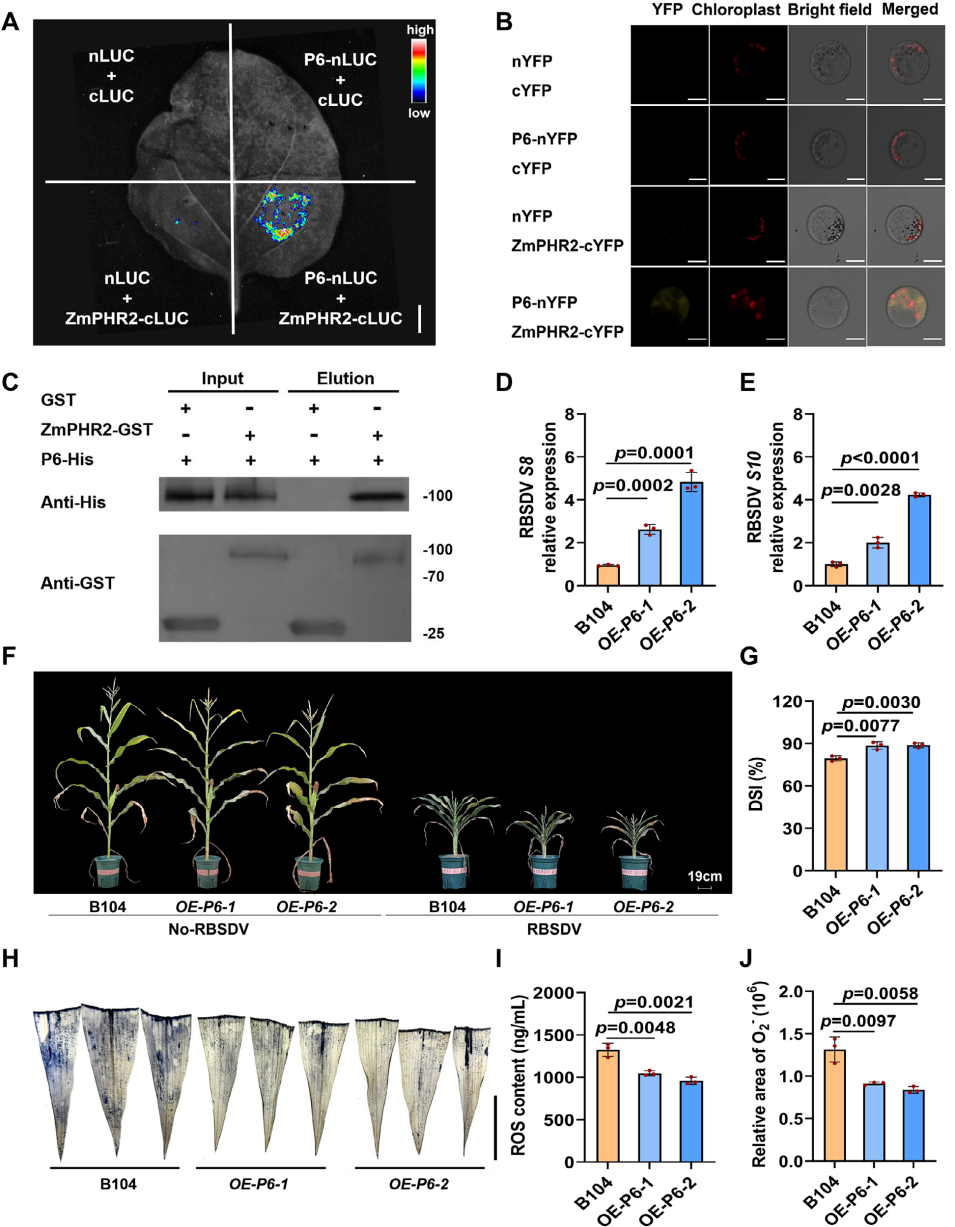
RBSDV P6 is an essential effector in RBSDV infection. (A) LCI assays in *Nicotiana benthamiana* leaves showing the interaction between ZmPHR2 and RBSDV P6. Scale bar = 1 cm. The nLUC/cLUC, P6‐nLUC/cLUC, nLUC/ZmPHR2‐cLUC constructs were separately infiltrated into the upper left, upper right and lower left parts of *Nicotiana benthamiana* leaves. The three co‐expressed samples were used as the negative controls in this assay. The P6‐nLUC/ZmPHR2‐cLUC constructs were infiltrated into the lower right part of *Nicotiana benthamiana* leaves, which were used as the experimental group. (B) BiFC assay shows that ZmPHR2 interact with RBSDV P6 in maize protoplast cells. RBSDV P6 was fused to the N‐terminal fragment of YFP (nYFP) and ZmPHR2 was fused to the C‐terminal fragment of YFP (cYFP). The interaction between cYFP and P6‐nYFP or nYFP and ZmPHR2‐cYFP serve as the negative controls. Scale bar = 10 µm. (C) Pull‐down assay shows that ZmPHR2 directly interacts with RBSDV P6 in vitro. (D, E) Relative expression levels of RBSDV *S8* (D) and *S10* (E) in B104 and RBSDV P6 overexpression plants after RBSDV inoculation. Data are means ± SD (*n* = 3 biologically independent samples with 6 plants per replicate). (F, G) MRDD phenotypes (F) and DSI values (G) of B104 and RBSDV P6 overexpression plants after inoculation. Scale bar = 19 cm. Data are means ± SD. from 3 biological replicates (*n* = 20 plants per replicate). (H) NBT staining of inoculated B104 plants and RBSDV P6 overexpression plants. Scale bar = 5 cm. (I, J) ROS content (I) and relative area of NBT staining (J) of B104 and RBSDV P6 overexpression plants. Data are means ± SD, (*n* = 3 biologically independent samples). Statistical significance was determined using Students’ *t*‐test. Exact *p* values are also directly marked on the (D, E, G, I and J).

Having identified P6 as a virulence effector that suppresses ROS, we hypothesized that it directly targets the ZmPHR2‐ZmISP complex to promote infection. To test this, we performed a yeast three‐hybrid (Y3H) assay, confirming that P6 strengthens the ZmPHR2‐ZmISP interaction (Figure 5A). Consistently, competitive pull‐down assays demonstrated a dose‐dependent enhancement of this binding by P6 (Figure 5B). We further evaluated the function of this enhanced interaction using LUC in *Nicotiana benthamiana*. Co‐expression of P6 with ZmPHR2 and ZmISP elevated luciferase activity but reduced NBT staining under white light, suggesting that P6 simultaneously inhibits ROS generation. Notably, under blue light, P6 no longer enhanced luciferase activity, and its suppressive effect on ROS accumulation was abolished, as indicated by NBT staining (Figure 5C–F). Together, these results demonstrate that RBSDV P6 facilitates infection by reinforcing the ZmPHR2‐ZmISP interaction and attenuating ROS burst, a mechanism effectively counteracted by blue light.

**FIGURE 5:**
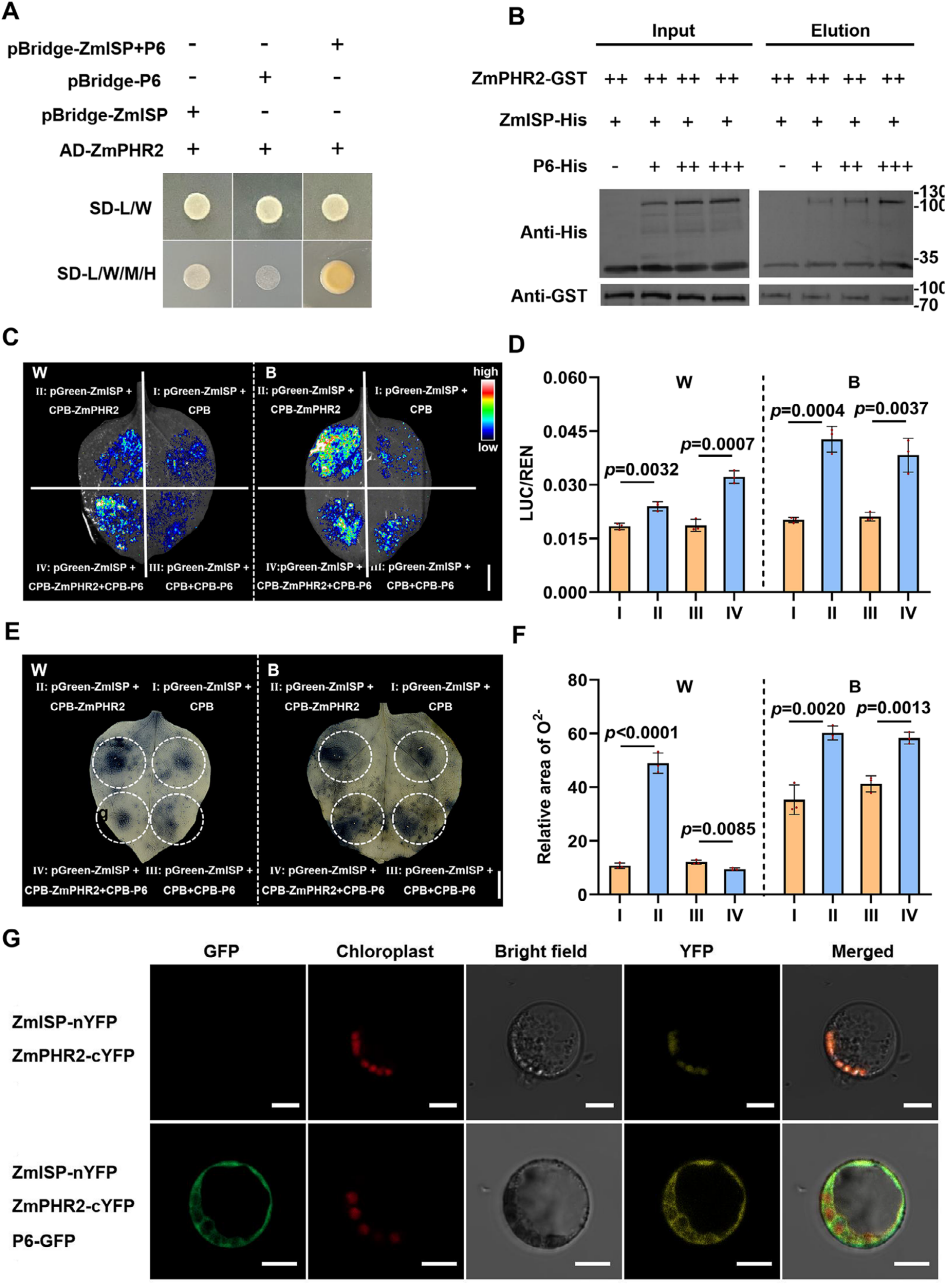
RBSDV P6 alters the subcellular co‐localization of ZmPHR2 and ZmISP. (A) Y3H assay shows that RBSDV P6 promotes the interaction between ZmPHR2 and ZmISP. (B) Competitive pull‐down assay to verify that RBSDV P6 promotes the interaction between ZmPHR2 and ZmISP. (C,D) Transient expression assay in *Nicotiana benthamiana* leaves showing ZmISP‐LUC activity upon co‐expression of ZmISP‐LUC and ZmPHR2 in the presence or absence of RBSDV P6 under white (W) and blue (B) light. Data are means ± SD (*n* = 3 biologically independent samples). Scale bar = 1 cm. (E,F) NBT staining assay revealing that RBSDV P6 inhibits ROS production under white light, whereas this suppression is abolished under blue light. Data are means ± SD (*n* = 3 biologically independent samples). Scale bar = 1 cm. (G) YFP fluorescence signals were used to observe the colocalization of ZmPHR2 and ZmISP, with chloroplast autofluorescence serving as the chloroplast marker. GFP fluorescence signals were used to observe the localization of RBSDV P6. Scale bar = 10 µm. Statistical significance was determined using Students’ *t*‐test.

Interestingly, our previous BiFC analysis detected the interaction between P6 and ZmPHR2 in the cytoplasm (Figure 4B), while the interaction between ZmPHR2 and ZmISP was observed in the chloroplasts (Figure 2D). We hypothesized that the RBSDV P6 protein might enhance the ZmPHR2‐ZmISP interaction by altering its subcellular context. Next, we examined the subcellular localization of each component. ZmPHR2 was localized at the plasma membrane and chloroplasts, ZmISP exclusively in chloroplasts, and RBSDV P6 at the plasma membrane (Figure S13). BiFC assays confirmed that the ZmPHR2‐ZmISP complex was localized in chloroplasts (Figure 2D). Strikingly, co‐expression of P6 resulted in detectable ZmPHR2‐ZmISP interaction signals in the cytoplasm in addition to chloroplasts (Figure 5G). Collectively, these data reveal that RBSDV P6 intensifies the physical association between ZmPHR2‐ZmISP and alters the subcellular distribution of this complex, which may serve as a core virulence strategy deployed by RBSDV during infection.

### 2.6 Conservation of the PHR2‐ISP Signaling Module in Rice

Viruses undergo continuous mutation to optimize their infectivity and adaptability in cereal crop hosts [52]. To investigate whether RBSDV has evolutionarily adapted to target conserved defense components in monocot hosts, we examined the functional presence of the PHR2‐ISP module in rice, a major natural host of the virus. Three RBSDV P6‐overexpression lines (*OE‐1^P6^*, *OE‐2^P6^* and *OE‐3^P6^*) in rice exhibited significant growth inhibition, including reduced plant height compared to the H9508 (Figures 6A and S14A), a phenotype consistent with that observed in RBSDV‐infected rice. This indicated that *P6* overexpression can perturb rice development and prompted us to test whether the rice PHR2‐ISP module is the conserved target. Phylogenetic analysis identified *OsPHR2* (*Os03g0343400*) and *OsISP* (*Os07g0556200*) as functional orthologs of *ZmPHR2* and *ZmISP* (Figure 6B,C). Overexpression of *OsPHR2* (*OE*‐*OsPHR2*) or *OsISP* (*OE*‐*OsISP*) in rice significantly reduced RBSDV accumulation upon inoculation, with reduced transcription levels of *S6*, *S8* and *S10* than in NIP (Figures 6D and S14B–E), suggesting that this module contributes to restricting RBSDV accumulation. We next examined the molecular basis of this functional conservation. The interaction between OsPHR2 and OsISP was confirmed by Y2H, LCI, and pull‐down assays, and BiFC assays revealed chloroplast localization (Figure 6E–G,K), consistent with the maize module. To determine whether P6 targets the rice PHR2‐ISP module to suppress immunity, we performed Y2H, LCI, and pull‐down assays, which confirmed that RBSDV P6 interacts with OsPHR2. BiFC assays localized this interaction to the cytoplasm (Figure 6H–J,L). Moreover, pull‐down assays further demonstrated that P6 also enhances the interaction between the OsPHR2 and OsISP complex in a dose‐dependent manner in rice (Figure 6M). Together, these findings reveal that RBSDV P6 has evolutionarily preserved the ability to target PHR2 orthologs across monocot species, underscoring a co‐evolutionary arms race centered on a central immune pathway.

**FIGURE 6:**
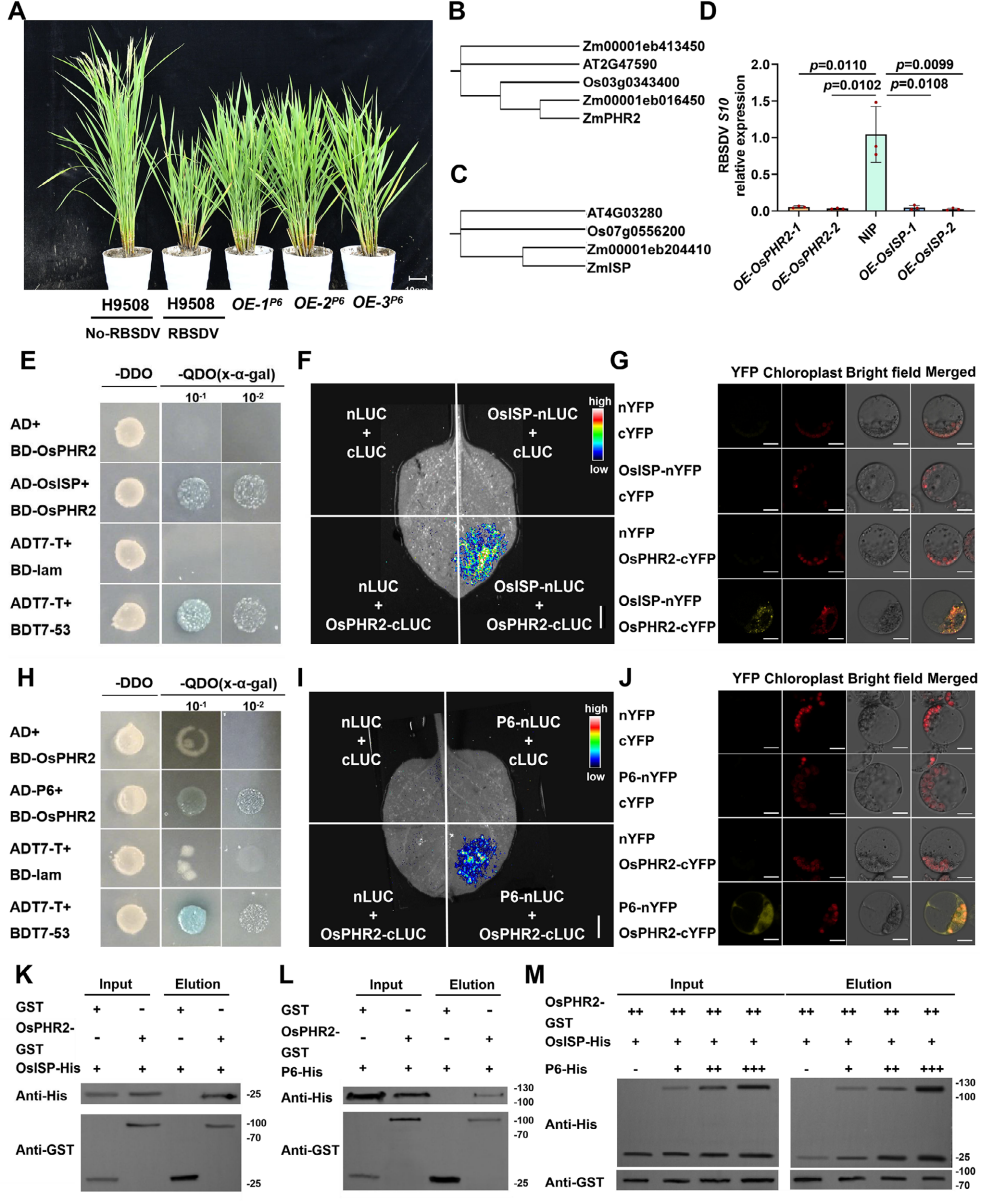
PHR2‐ISP module was conserved in rice. (A) Phenotypic analysis of H9508 and RBSDV P6 overexpression plants in rice. Scale bar = 10 cm. (B‐C) Phylogenetic analysis of *ZmPHR2* (B) and *ZmISP* (C) proteins among *Arabidopsis*, rice and maize. (D) Relative transcript level of RBSDV *S10* in NIP, *OsPHR2* and *OsISP* overexpression lines after RBSDV inoculation. (Data are means ± SD, *n* = 3 biologically independent samples). (E–G) Verification of the interaction between OsPHR2 and OsISP using Y2H (E), LCI (F), and BiFC assays (G). (H–J) Verification of the interaction between OsPHR2 and RBSDV P6 using Y2H (H), LCI (I), and BiFC assays (J). Scale bar = 1 cm in (F) and (I); Scale bar = 10 µm in (G) and (J). (K, L) Pull‐down assays to verify the interaction between OsPHR2 and OsISP (K), RBSDV P6 and OsPHR2 (L). (M) Competitive pull‐down assay to verify that RBSDV P6 enhances the interaction between OsPHR2 and OsISP.

## 3 Discussion

RBSDV poses a significant economic threat to global cereal production by causing diseases such as MRDD and rice black‐streaked dwarf disease (RBSDD) [29, 53, 54, 55], making the dissection of RBSDV‐host antiviral interactions critical for crop protection. While several key resistance mechanisms have been identified, only a few genes such as *OsGLK1* [56] and *OsAP47* [55] in rice, as well as *ZmGDlα‐hel* [35, 36] and *ZmGLK36* [37] from maize, have been functionally characterized so far. They mainly participate in plant hormone pathways, such as gibberellins and jasmonic acids [1, 37, 55, 56]. Maize resistance to RBSDV is a quantitative trait, and it is necessary to discover new resistance genes. Here, we report the identification of a novel antiviral module activated by blue light, in which the photoreceptor PHR2 interacts with the Fe‐S subunit (ISP) of the cytochrome b6/f complex within chloroplasts (Figure 7). Our findings reveal a previously unrecognized link between photoreceptor signaling and antiviral defense in chloroplasts. While previous studies in *Arabidopsis* have established that CRY1 mediates blue light‐dependent stomatal opening and pathogen‐induced stomatal closure, thereby regulating stomatal immunity and acts as a dual‐function switch coordinating photosynthesis and PTI immunity [14], our work demonstrates a distinct mechanism. We show that PHR2, in conjunction with ISP, is associated with chloroplast integrity and facilitates ROS accumulation to confer viral resistance. It is noteworthy that the P6‐PHR2‐ISP regulatory module is functionally conserved across major monocot species (Figures 6 and S14), suggesting that PHR2 and ISP have undergone a long‐term co‐evolutionary process with RBSDV, which may enable sustained antiviral defense in cereal crops. This evolutionary conservation underscores the potential of engineering PHR2‐ISP interactions to develop resistance against RBSDV and related viruses in cereal crops. We therefore propose that in dynamically changing environments, blue light receptors function as a central regulatory switch, enabling plants to optimally balance photosynthetic efficiency and immune responses, thus harmonizing growth and defense.

**FIGURE 7:**
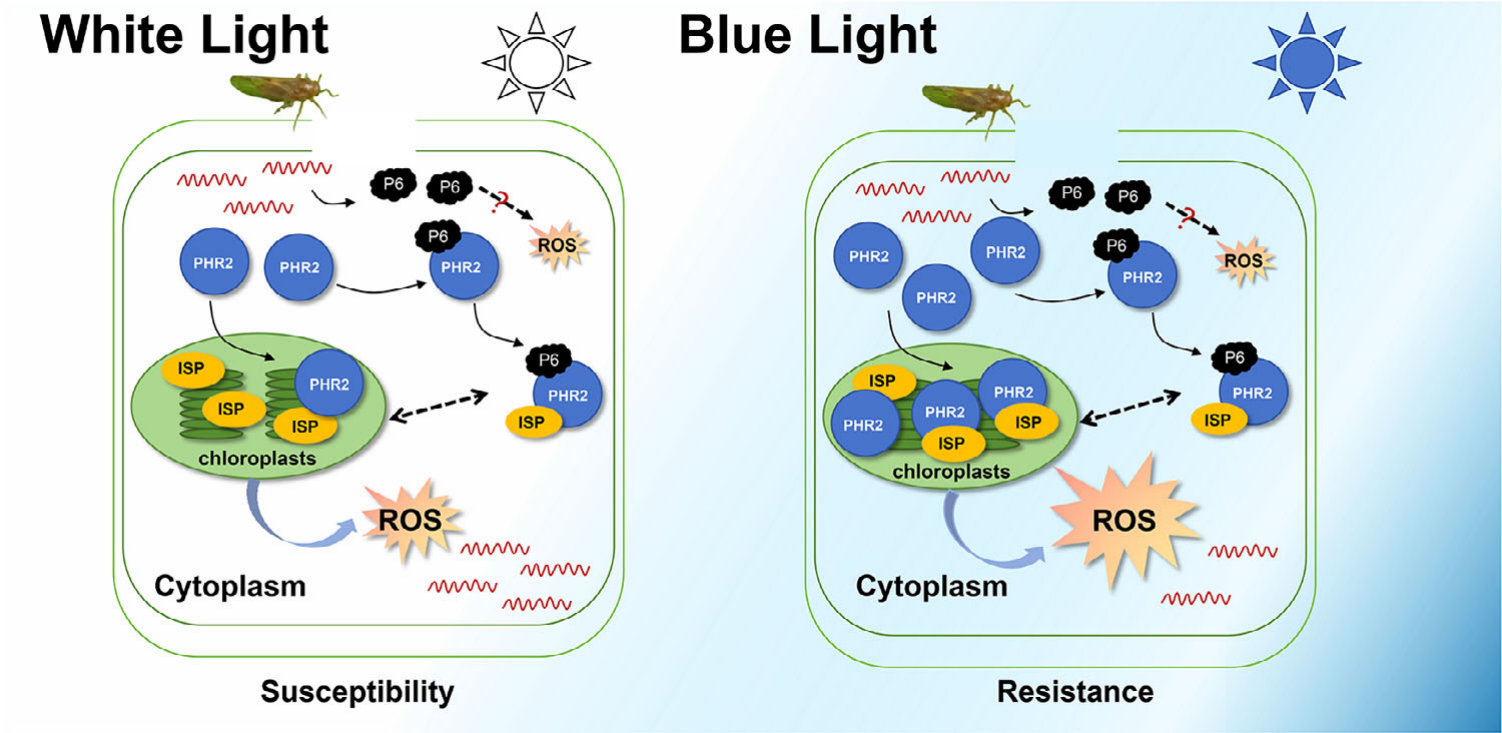
P6‐PHR2‐ISP module‐mediated resistance to maize rough dwarf disease. RBSDV P6 binds to PHR2 and enhances its interaction with ISP, resulting in detectable PHR2‐ISP interaction signals in the cytoplasm and reduced ROS production. Blue light strongly induces *PHR2* expression. Under constitutive RBSDV P6 expression, increased PHR2 abundance promotes its chloroplast localization and binding to ISP, leading to elevated ROS levels. This enhanced ROS production strengthens cereal crop resistance to RBSDV. The dashed line with a question mark indicates a possible re‐localization‐independent pathway by which P6 may suppress ROS; whether this pathway exists remains to be determined.

To our knowledge, blue light and its receptors play a crucial role in regulating plant growth and enhancing the disease resistance of bacteria or fungi [1, 14]. They can integrate internal and external signals. Recently, they have been proposed as promising targets for improving agronomic traits in economically important crops such as wheat and rice [57, 58, 59]. In this study, we demonstrate that blue light and its receptor ZmPHR2 also play an important role in antiviral immunity by recruiting ZmISP [44, 60]. After blue light irradiation, ZmPHR2 expression and protein accumulation are elevated, enhancing resistance to RBSDV (Figures 1E and S4C). Within chloroplasts, ZmPHR2 mediates ROS accumulation through ZmISP to confer resistance against RBSDV (Figures 2A–D and 3A–J; Figure S10). To our knowledge, this is the first report demonstrating that a blue light receptor mediates ROS‐dependent antiviral immunity, providing novel targets for the genetic improvement of RBSDV resistance in cereals.

Our data show that RBSDV P6 enhances the ZmPHR2‐ZmISP interaction, alters their subcellular distribution, and correlates with suppressed ROS accumulation (Figure 5G). Given the dual localization of ZmPHR2 in both the cytoplasm and chloroplasts, one model posits that P6 binds ZmPHR2 in the cytosol, preventing its chloroplast import and consequently retaining ZmISP precursors bound to ZmPHR2 in the cytoplasm, thereby blocking ZmISP import. An alternative model is that P6 promotes the export of already‐imported ZmPHR2‐ZmISP complexes from chloroplasts through an unknown mechanism. We favor the precursor sequestration model but cannot fully exclude minor extraction of mature ZmISP. Notably, wheat stripe rust effectors Pst_4, and Pst_5 similarly target the chloroplast import step, they bind TaISP precursors in the cytosol to block import and suppress ROS [44]. Although the molecular details differ, both strategies converge on disrupting chloroplast precursor import, underscoring the centrality of this node in plant immunity. If the ZmPHR2 retention model is correct, aberrant cytosolic accumulation of ZmPHR2‐ZmISP complexes may trigger disrupted retrograde signaling, offering a mechanistic explanation for the severe chloroplast damage documented in RBSDV‐infected maize [61]. The present study objectively reports the P6‐induced re‐localization phenotype. Further elucidation of the underlying mechanism will require cTP truncation mutants, in vitro chloroplast import assays, and biochemical analysis of the P6‐ZmPHR2 complex. We acknowledge that the current data do not establish whether P6‐induced re‐localization is sufficient to account for the observed ROS suppression. Therefore, a re‐localization independent pathway for ROS inhibition remains a possibility. Future work combining quantitative time‐lapse imaging and single‐cell analyses will be required to resolve this uncertainty.

Our study also reveals the superior therapeutic efficacy of blue light against RBSDV compared to white light (Figure S1). Genetic evidence indicates that ZmPHR2 significantly reduces viral RNA accumulation (Figures 1K and S6C,D), suggesting that blue light illumination enhances antiviral immunity by activating ZmPHR2‐mediated ROS signaling. Furthermore, RBSDV increases ZmPHR2 RNA and protein accumulation (Figures 1F and S4B). Consequently, we hypothesize that an unidentified upstream factor may sense blue light or viral infection and transduce the signal to ZmPHR2, either directly or indirectly, to activate downstream defense responses. Identifying this sensor is a critical goal for future research. Given the widespread use of blue light in agricultural settings such as greenhouses, our findings are of practical relevance. This study provides evidence that blue light modulates ZmPHR2‐mediated ROS signaling to regulate antiviral defense and plant development. Our results raise the possibility of blue light‐mediated antiviral defense in agriculture, but practical application needs optimization of light conditions and field‐based validation across multiple environments. Our results thus offer novel insights into blue light application and the ZmPHR2‐regulated antiviral pathway. Based on these findings, we propose several actionable strategies to advance applications. First, identifying upstream light signaling regulators of ZmPHR2 could enable precise, light‐controlled resistance pathways. Second, applying single‐cell sequencing to dissect chloroplast‐cytoplasm communication during RBSDV infection may reveal novel antiviral targets. Third, synthetic approaches such as engineering light‐responsive promoters could allow on‐demand activation of defense, balancing immunity, and growth. Fourth, resolving the P6‐ZmPHR2‐ZmISP regulatory mechanism through cTP truncation mutants and in vitro chloroplast import assays will clarify whether P6 targets precursor or mature ZmISP and establish the causal link to ROS suppression. Fifth, through techniques such as gene editing combined with doubled haploid breeding, the PHR2 and ISP pathways can be engineered to enhance crop resistance to viruses and photosynthetic capabilities. Together, these strategies could translate mechanistic insights into optimized agricultural practices.

## 4 Methods

### 4.1 Plant Materials and Growth Conditions

Maize inbred lines (B73 and B104) and transgenic lines were used in this study. The *ZmPHR2* knockout lines, overexpression lines and *ZmISP* overexpression lines were generated in the RBSDV‐susceptible maize inbred line B104. The *zmphr2* and *zmisp* mutants were isolated from an EMS‐mutagenized maize library [62], introgressed into B73 with twice backcrosses and then selfed for phenotypic analysis. The wild‐type (WT) control used in this study was derived from non‐mutant siblings of the transgenic plants. Rice cultivar H9508 and Nipponbare (NIP) were employed as the recipient for rice transgenic plant generation. Maize seedlings and transgenic rice plants were grown in the experimental fields of the Chinese Academy of Agricultural Sciences in Beijing (N40°13′54″, E116°33′50″) and Sanya (N18°23′23″, E109°11′48″), Hainan Province during natural growing seasons. Transgenic lines were propagated to obtain homozygous plants, which were self‐pollinated for seed propagation and subsequently used for RBSDV inoculation and phenotypic analysis.

For light treatment experiments, maize seedlings of inbred line B73 were sown in 72‐well trays filled with sterilized substrate and cultivated at 28°C under a 16 h light / 8 h dark photoperiod for 10 days. Seedlings at the three‐leaf stage were then exposed to either white (W, 34 µmol m^−2^s^−1^) or blue (B, λ_p__e__a__k_ = 480 nm, 31 µmol m^−2^s^−1^) light for 2 h. All light parameters were quantified using a HiPoint HR‐350 spectrometer prior to treatment initiation.

### 4.2 Vector Constructs and Plant Transformation

To generate the transgenic vectors, the coding sequences of *ZmPHR2* and *ZmISP* were fused with the green fluorescent protein (GFP) coding sequence, respectively. The full‐length coding regions of *ZmPHR2* and *ZmISP* were amplified using gene‐specific primers, and the resulting PCR fragments were in‐frame ligated into the *CUB‐EGFP‐3×Flag* and *CUB‐EGFP‐6×Myc* vectors at *Bst*EII and *Bam*HI restriction sites, respectively [63]. These recombinant constructs were introduced into immature embryo of the maize inbred line B104 through *Agrobacterium*‐mediated transformation, resulting in eight and three independent *ZmPHR2*‐GFP and *ZmISP*‐GFP transgenic lines, respectively. Two homozygous lines from each construct were selected for subsequent experimental analyses. For the generation of *ZmPHR2* knockout lines, the CRISPR/Cas9 construct was constructed as previously described [64]. Three guide RNAs (gRNAs) targeting *ZmPHR2* were designed based on the reference genome sequence of maize inbred lines B73 and B104 using the CRISPR‐P web tool (http://cbi.hzau.edu.cn/crispr/). The target sequences of the designed gRNAs are provided in Figure S5B. To construct the RBSDV P6 transgenic vector, the coding region of the RBSDV *S6* gene (encoding the P6 protein) was amplified and cloned into the *CPB‐EGFP‐6×Myc* vector at the *BstE*II restriction site. All constructs were verified by Sanger sequencing to ensure sequence accuracy, and the sequences of all primers used for vector construction are listed in Table S1.

### 4.3 Artificial Inoculation of RBSDV and Phenotypic Evaluation

Maize seedlings at the three‐leaf (V3) growth stage were artificially inoculated with RBSDV using viruliferous small brown planthoppers (SBPH). Seedlings in the control group were exposed to non‐viruliferous (virus‐free) SBPH under identical conditions. After 48 h of inoculation, all SBPH were carefully removed to avoid excessive feeding damage and secondary virus transmission. Inoculation and control plants were then transplanted to the experimental field in Beijing for subsequent growth. MRDD symptoms were evaluated at the silking stage (R1 stage) using a modified 0–4 disease rating scale, as previously described (0 = highly resistant, 4 = highly susceptible) [55]. The disease severity index (DSI) was calculated using the following formula: DSI (%)  = Σ (disease rating score × number of plants with each score) / (maximum disease rating score × total number of plants rated in the line) × 100.

### 4.4 RNA Extraction and RT‐qPCR Analysis

To investigate gene expression patterns in response to RBSDV infection, V3‐stage maize seedlings were inoculated with viruliferous SBPH as described previously, followed by incubation under white or blue light. Leaf samples were collected at 0, 6, and 12 days post inoculation (dpi). For evaluating the effect of light quality on gene expression, V2‐stage seedlings were exposed to white or blue light for 2 h, with leaf tissues harvested at 0, 0.25, 0.5, 1, and 2 h post‐treatment. For the combined RBSDV‐light treatment, RBSDV‐inoculated seedlings were subjected to white or blue light irradiation for 2 h, and leaf samples were collected at 0, 6, and 12 dpi. Additionally, various tissues from wild‐type maize seedlings and adult plants were sampled to determine the tissue‐specific expression profiles of target genes. Total RNA was extracted from all collected samples using the RNA‐easy Isolation Reagent (Cat 701‐01, Vazyme) following the manufacturer's protocol. Complementary DNA (cDNA) was synthesized from 1 µg of total RNA using the FastQuant Reverse Transcription Kit (Cat KR106, Tiangen). Quantitative real‐time PCR (RT‐qPCR) was performed on a CFX Connect Real‐Time PCR System (Bio‐Rad, USA) using SuperReal PreMix Plus (SYBR Green) (Cat FP205, Tiangen) according to the manufacturer's instructions. *ZmUbiquitin* was used as the internal reference gene, and relative gene expression levels were calculated using the 2^^(−ΔΔCt)^ method [65]. All data are presented as the means ± SD from three independent biological replicates. Primers used for RT‐qPCR are provided in Table S1.

### 4.5 Sequence Alignment and Phylogenetic Analysis

Full‐length amino acid sequence of ZmCRY family members in maize were retrieved from the Gramene (http://www.gramene.org/) and NCBI (https://www.ncbi.nlm.nih.gov/) databases. Multiple sequence alignment was performed using ClustalW with default parameters. A phylogenetic tree was subsequently constructed in MEGA11 using the Neighbor‐Joining method, based on the aligned amino acid sequences, with bootstrap testing (1000 replicates) [66].

### 4.6 ROS Detection

To assess ROS levels, the third leaves of V2‐stage maize seedlings from the following genotypes: B73, B104, *ZmPHR2* knockout lines, *ZmPHR2* overexpression lines, *ZmISP* overexpression lines, and *zmisp* mutants, were harvested after a 2‐h exposure to either white light (W, 34 µmol m^−^^2^s^−^^1^) or blue light (B, 31 µmol m^−^^2^s^−^^1^). Each treatment included three biological replicates. The harvested leaf tissues were stained using a Plant ROS Detection Kit (Cat G4816, Solarbio) according to the manufacturer's protocol. Briefly, excised leaves were fully immersed in the staining solution and incubated overnight at room temperature under dark conditions. Following the appearance of distinct blue‐green spots, the leaves were rinsed 2–3 times with distilled water and subsequently decolorized in absolute ethanol at room temperature until the tissues turned completely yellow‐white. Quantitative ROS levels were further determined using a ROS ELISA Detection Kit (Cat CB10054, Keyibo Biological, Shanghai) according to the manufacturer's instructions. The stained areas and corresponding ROS levels were quantified using Image J software 1.54, and the results data were visualized with GraphPad Prism 8.0.

### 4.7 NAC Treatment

V3‐stage maize seedlings of B104, *OE*‐*ZmPHR2* and *OE*‐*ZmISP* were sprayed with water (CK) or 40 mM N‐acetyl‐L‐cysteine (NAC) prior 2 h to RBSDV inoculation. NAC or water was sprayed every 2 days after inoculation until sample collection at 12 days post inoculation. Each treatment included three biological replicates with six plants per replicate. The NBT staining areas were quantified using Image J, and the data were visualized with GraphPad Prism 8.0.

### 4.8 Histological Analysis

Chloroplast ultrastructure in leaves of B73, B104, *ZmPHR2* knockout lines, and *zmisp* mutants was examined using transmission electron microscopy (TEM). Fresh leaf segments (1 cm × 1 cm) were vacuum‐infiltrated and fixed in 2.5% glutaraldehyde at 4°C for 4–6 h, followed by three washes with 0.1 M phosphate buffer (pH 7.2). Samples were then post‐fixed in 1% osmium tetroxide at 4°C for 2 h and washed again with the same buffer. Subsequently, the samples were dehydrated through a graded ethanol series (30%, 50%, 70%, 95%, and 100%), infiltrated with Spurr's resin using acetone‐resin gradients, and polymerized at 60°C for 24 h. Ultrathin sections (70 nm) were prepared from the polymerized blocks, stained with uranyl acetate and lead citrate, and observed under a Hitachi HT7700 transmission electron microscope.

### 4.9 RNA‐seq Analysis

Leaf samples were collected from V3‐stage seedlings of the maize inbred line B104, along with *ZmPHR2* and *ZmISP* overexpression lines. Total RNA was extracted, and RNA sequencing was performed by Beijing Allwegene, with three biological replicates per sample. Sequencing libraries were prepared and sequenced on an Illumina platform following standard protocols. Raw sequencing reads were processed using CASAVA for base calling, and subsequent quality control was applied to generate clean reads. Clean reads were then aligned to the reference genome, and read counts for each gene were obtained. Differential expression analysis was conducted using appropriate statistical models. *P*‐values were calculated based on the model hypothesis, and the false discovery rate (FDR) was controlled using multiple testing correction. Genes exhibiting |log_2_ (fold change) | ≥ 1 and FDR < 0.05 were defined as differentially expressed genes (DEGs). Functional enrichment analysis was carried out using Go‐seq for Gene Ontology (GO) terms and KOBAS (2.0) for KEGG pathways [67].

### 4.10 Protein Extraction and Immunoblotting

For western blotting, leaf tissues from V3‐stage seedlings were ground to a fine powder in liquid nitrogen and homogenized in pre‐chilled RIPA Lysis Buffer (Cat CW2333S, Kangwei). The homogenate was centrifuged, and the supernatant was collected for total protein quantification using the Bradford assay. Proteins were separated by SDS‐PAGE and transferred onto a PVDF membrane for immunoblotting. Anti‐ZmPHR2 (1:1000, PHY6432A, PhytoAB) and Anti‐OsPetC antibodies (1:1000, PHY4657A, PhytoAB) were used for the detection of ZmPHR2 and ZmISP, respectively. Anti‐GFP antibody (1:2000, Cat ab6556, abcam) was used for the detection of GFP‐tagged proteins. Actin (1:5000, Cat AC053, AB‐clonal) was used as a loading control. HRP‐conjugated Goat anti‐Rabbit IgG (H+L) (1:10000, Cat AS014, AB‐clonal) was used as the secondary antibody. Protein signals were visualized using the Fujifilm LAS‐3000 imaging system.

### 4.11 Subcellular Localization Assays

To determine the subcellular localization of ZmPHR2, ZmISP, and RBSDV P6 proteins, the full‐length coding sequences (without stop codons) were amplified from B73 cDNA and cloned into the *pAN580* vector using the *Bam*HI restriction site. The FM 4–64 (Cat T3166, Thermo) and chloroplast autofluorescence were used as membrane and chloroplast markers, respectively. The resulting fusion constructs and markers were co‐transfected into maize protoplasts via polyethylene glycol (PEG)‐mediated transformation, as previously described [68]. After transfection, protoplasts were cultured in the dark at 25°C for 12–18 h, and fluorescence signals were visualized using an LSM980 confocal laser‐scanning microscope. All primers used for vector construction are listed in Table S1.

### 4.12 Y2H And Y3H Assays

For Y2H assay, the full‐length coding regions of *ZmPHR2*, *OsPHR2*, *ZmISP*, and *OsISP* were cloned into the *pGBKT7* and *pGADT7* vectors using the *Eco*RI restriction site. Additionally, coding sequences of RBSDV proteins (P1‐P10) were inserted into the *pGADT7* vector using *Eco*RI and *Bam*HI restriction sites. Plasmid pairs were co‐transformed into the Y2H Gold yeast strain (Cat YC1002, Weidi) and plated on SD/‐Leu/‐Trp medium. Positive clones were subsequently screened on SD/‐Leu/‐Trp/‐His/‐Ade medium supplemented with X‐α‐gal. The *pGADT7‐T*/*pGBKT7‐Lam* and *pGADT7‐T*/*pGBKT7‐53* pairs were included as negative and positive controls, respectively. All procedures were performed following the manufacturer's instructions (Clontech Yeast Protocols Handbook).

For Y3H assay, the coding sequences of *ZmISP* and *RBSDV P6* were cloned into the *pBridge* vector to generate *pBridge‐ZmISP* and *pBridge‐P6*, respectively. The *RBSDV P6* coding sequence was further inserted into *pBridge‐ZmISP* via the *Bgl*II site to generate the *pBridge‐ZmISP‐P6* construct. The *AD‐ZmPHR2* plasmid was co‐transformed with each *pBridge* construct into Y2H Gold yeast. Transformants were selected on SD/‐Leu/‐Trp medium and subsequently screened for protein interaction on SD/‐Leu/‐Trp/‐His/‐Met selection medium. All primers used in this study are listed in Table S1.

### 4.13 LCI, LUC and BiFC Assays

For LCI assay, the full‐length coding sequences of *ZmISP*, *RBSDV P6*, and *OsISP* were cloned into the *pCAMBIA1300*‐*nLUC* vector, while *ZmPHR2* and *OsPHR2* were inserted into *pCAMBIA1300‐cLUC*. All constructs were transformed into *Agrobacterium tumefaciens* strain GV3101. Bacterial cultures were grown for 2–3 days at 28°C, resuspended in infiltration buffer, and co‐infiltrated into *Nicotiana benthamiana* leaves. After incubation at 25°C for 2–3 days, the infiltrated leaf areas were sprayed with 20 mg/mL potassium luciferin (Promega), and luminescence signals were captured using a Night SHADE LB 985 imaging system (Berthold, Germany). For LUC assay, the full‐length coding sequence of *ZmISP*, was cloned into *pGreen0800‐35s* at the *BamH*I restriction site, *ZmPHR2* into *CPB‐EGFP‐3×Flag* with the *BstE*II restriction site, and P6 into *CPB‐EGFP‐6×Myc* using the *BstE*II restriction site. These constructs were similarly introduced into *Nicotiana benthamiana* leaves via *Agrobacterium*‐mediated transient expression. Luminescence detection was performed as described above. For BiFC assay, the coding sequences of *RBSDV P6*, *ZmISP*, and *OsISP* were cloned into *pAN580*‐*nYFP*, *ZmPHR2* and *OsPHR2* were inserted into *pAN580*‐*cYFP*. Construct pairs were co‐transfected into maize protoplasts, and YFP fluorescence was observed after 12–18 h using a Zeiss LSM980 confocal microscope. All primers are listed in Table S1.

### 4.14 In Vitro Pull‐Down Assays

For recombinant protein expression, the coding sequences of *ZmPHR2* and *ZmISP* were cloned into *pET‐41a* and *pCzn1* vectors, generating ZmPHR2‐GST and ZmISP‐His fusion construct, respectively. Each construct was transformed into *E. coli* strain BL21 (DE3) competent cells (TransGen Biotech, China). A single positive colony was inoculated into 2 mL of LB medium containing ampicillin and cultured at 37°C until OD_600_ reached 0.5. This starter culture was then transferred into 100 mL of fresh LB medium and incubated at 25°C with shaking until OD_600_ reached 0.5. Protein expression was induced by adding 1 mM IPTG (final concentration), followed by continued incubation for 4–6 h. Cells were harvested by centrifugation at 4000*×g* for 10 min, resuspended in lysis buffer, and lysed via sonication on ice. The lysate was centrifuged at 8000*×g* for 20 min, and the supernatant was incubated with either GST agarose beads or Ni‐NTA His‐Bind resin (Novagen) according to the manufacturers’ protocols for protein purification.

For the pull‐down assay, purified ZmPHR2‐GST was incubated with ZmISP‐His cell lysate at 4°C for 1 h with gentle rotation. Beads were washed three times with protein extraction buffer, and bound proteins were eluted by boiling in SDS loading buffer. Samples were separated by 10% SDS‐PAGE and analyzed by immunoblotting using Anti‐GST (1:5000, Cat 10079, zoonbio) and Anti‐His (1:4000, Cat 10010, zoonbio) antibodies.

### 4.15 Statistics and Reproducibility

All statistical calculations and graph were performed using GraphPad Prism 8.0. All raw data were used directly without additional mathematical transformations. Each experiment included a minimum of 3 biological or technical replicates; the exact sample quantity and definition of replicate categories are clearly stated in each figure legend. All quantitative data are presented as mean ± SD (standard deviation), as specified for individual figures. Student's *t*‐test was used to compare pairwise differences between experimental groups. Two forms of marking were used to indicate statistical results: asterisks were only assigned to significant differences, with * indicating *p* < 0.05 and ** indicating *p* < 0.01; no markers were added for comparisons with *p* > 0.05. In addition to asterisk labeling, exact *p* values were printed directly on some graph panels. The statistical method applied to each dataset is detailed in the corresponding figure legends.

## Author Contributions

R. C. performed most of the experiments. Y. T. wrote the manuscript and performed a portion of the experiments. X. W. performed a yeast two‐hybrid library screening assay. Z. X, J. W. and X. L. designed the research. G. L. and J. L. characterized the genotypes and phenotypes of the edited lines. X. L, C. M, X. L, Z. Z, J. H, D. S. and Z. H. analyzed the data. M. L, D. Z. and H. Y. helped correct the manuscript. All the authors have read and approved the final manuscript.

## Conflicts of Interest

The authors declare no conflicts of interest.

## Supporting information




**Supporting File 1**: advs78050‐sup‐0001‐SuppMat.docx.

**Supporting File 2**: advs78050‐sup‐0002‐SuppMat.xlsx.

**Supporting File 3**: advs78050‐sup‐0003‐SuppMat.docx.

**Supporting File 4**: advs78050‐sup‐0004‐SuppMat.xlsx.

## Data Availability

Data supporting the findings are presented in the main text or the supplementary materials. Any additional data are available from the corresponding author. All real‐time PCR and other quantitative analysis were repeated at least three times. The datasets generated in this study were provided in the article. Source data are provided with this paper.
