# Supplementary material for: Blue Light Receptor PHR2‐Mediated Spectrum Antiviral Immunity
Source: Adv Sci (Weinh). 2026 Sep 30:e78050. Online ahead of print. doi: 10.1002/advs.78050 (PMC13626285; doi:10.1002/advs.78050)
Supplement: Supplementary file 1 [file ADVS-9999-e78050-s004.docx]

**Supplementary Figure Legends**

**Figure S1. Blue light activates SA, JA, and ROS pathways simultaneously.**

(A) ROS detection in B73 leaves exposed to white and blue light, respectively. Scale bar = 2 cm. (B) NBT staining areas of leaves under white and blue light treatments (n = 3 biologically independent samples). (C) ROS content of leaves under white and blue light treatments (n = 3 biologically independent samples, three plants per biological replicates). (D-G) Blue light-mediated induction of key genes in SA and JA signaling pathways. Relative expression levels of *ZmNPR1* (D), *ZmEDS1* (E), *ZmAOC* (F) and *ZmAOS* (G) under blue and white light treatments for 2 h. Data are means ± SD (n = 3 biologically independent samples with six plants per biological replicate). Statistical significance was determined using Students’ ***t***-test. Exact *p* values are directly marked on the (B and C). * *p* < 0.05, ** *p* < 0.01 are used to label significant differences in (D-G); no labels are shown for comparisons with *p* > 0.05.

**Figure S2. Phylogenetic analysis and blue light-responsive expression patterns of *ZmCRY* family members in maize.**

(A) Phylogenetic analysis of *ZmCRY* family members in maize. The left panel shows the phylogenetic tree constructed for ZmCRY proteins. The right panel depicts the domain composition of each ZmCRY protein, with different colors representing distinct domains: green for low complexity region, yellow for Pfam: DNA photolyase, pink for Pfam: FAD binding 7, teal for Pfam: Cryptochrome C, and red for SCOP: driqra1. The scale bar at the bottom indicates the length of the protein sequences (in amino acid residues). (B-I) Relative expression levels of *ZmCRY1a1* (B), *ZmCRY1a2* (C), *ZmCRY1b* (D), *ZmCRY2* (E), *ZmCRY3* (F), *ZmPHR1* (G), *ZmCRYSF* (H), and *ZmCRYPHO* (I) at different time points under white and blue light treatments. Data are means ± SD. (n = 3 biologically independent samples with six plants per biological replicate). Statistical significance was determined using Students’ ***t***-test. * *p* < 0.05, ** *p* < 0.01 are used to label significant differences in (B-I); no labels are shown for comparisons with *p* > 0.05.

**Figure S3. Expression patterns of *ZmCRY* family members in maize after RBSDV inoculation.**

(A-H) Relative expression levels of *ZmCRY1a1* (A), *ZmCRY1a2* (B), *ZmCRY1b* (C), *ZmCRY2* (D), *ZmCRY3* (E), *ZmPHR1* (F), *ZmCRYSF* (G), and *ZmCRYPHO* (H) under RBSDV inoculation (Y) or non-inoculation (N) at 0, 6 and 12 days post inoculation. Data are means ± SD. (n = 3 biologically independent samples with six plants per biological replicate). Statistical significance was determined using Students’ ***t***-test (* *p* < 0.05, ** *p* < 0.01 are used to label significant differences; no labels are shown for comparisons with *p* > 0.05.).

**Figure S4. Identification, expression pattern and protein expression levels of *ZmPHR2* under blue light treatment at different time points after RBSDV inoculation.**

(A) RT-qPCR analysis of *ZmPHR2* in various tissues in B73. Data are means ± SD (n = 3 biological replicates). (B) Immunoblot analysis of ZmPHR2 protein accumulation in maize under RBSDV inoculation (Y) or non-inoculation (N) at 0, 6 and 12 days post inoculation. Actin was used as an internal control, and relative protein levels were normalized to Y 0 d / N 0 d (set to 1.0), quantified using Image J, and shown below the blots. Cropped from original blots with left-right order of the Y and N groups adjusted for presentation. Full-length blots are provided in Source Data. (C) Immunoblot analysis of ZmPHR2 protein accumulation in maize exposed to blue (B 0 h, B 0.25 h, B 0.5 h, B 1 h, B 2 h) and white light (W 0 h, W 0.25 h, W 0.5 h, W 1 h, W 2 h). Actin was used as an internal control, and relative protein levels were normalized to B 0 h / W 0 h (set to 1.0), quantified using Image J, and shown below the blots.

**Figure S5. Phenotypic characterization and disease resistance evaluation of *zmphr2* mutant and *ZmPHR2* knockout mutants.**

(A) *ZmPHR2* gene structure in B73 and *zmphr2* mutant. DNA photolyase domain and nucleotide polymorphism responsible for *zmphr2* mutation are indicated by red box. (B) Sequencing analysis showing mutations in the CRISPR/Cas9-generated *ZmPHR2* knockout mutants. (C) MRDD phenotypes of B73 and *zmphr2* mutants under indicated treatments. No-RBSDV, plants not inoculated with RBSDV; RBSDV, plants artificially inoculated with RBSDV at the V3 stage. Scale bar = 15cm. (D-F) Relative expression levels of RBSDV *S6* (D), *S8* (E), and *S10* (F) in B73 and *zmphr2* mutants. Data are means ± SD (n = 3 biologically independent samples with six plants per biological replicate). (G) DSI values of B73 and *zmphr2* mutants after RBSDV inoculation. Data are means ± SD (n = 3 biologically independent samples with 20 plants per biological replicate). (H, I) The relative expression level of RBSDV *S6* (H) and *S8* (I) in B104 and *ZmPHR2* knockout mutants after RBSDV inoculation. Data are means ± SD (n = 3 biologically independent samples with six plants per biological replicate). (J-L) The plant height (J)**,** ear height (K) and relative chlorophyll content (L) of B73 (n = 28) and *zmphr2* mutants (n = 34). Data are means ± SD. (M-O) Plant height (M), ear height (N) and relative chlorophyll content (O) of B104 (n = 25), *KO-ZmPHR2-1* (n = 25) and *KO-ZmPHR2-2* (n = 25) knockout mutants. Data are means ± SD. Statistical significance was determined by Students’ ***t***-test. Exact *p* values are directly marked on the (D-O).

**Figure S6. Identification of *ZmPHR2* overexpression transgenic plants.**

(A) Western blot analysis showing the expression of *ZmPHR2* overexpression lines. Anti-GFP antibody was used to detect ZmPHR2 protein and Actin was used as an internal control. (B) The relative expression level of *ZmPHR2* in B104 and *ZmPHR2* overexpression plants (Data are means ± SD, n = 3 biologically independent samples). (C, D) The relative expression level of RBSDV *S6* (C) and *S8* (D) in B104 and *ZmPHR2* overexpression plants after RBSDV inoculation. Data are means ± SD (n = 3 biologically independent samples with six plants per biological replicate). (E-G) Plant height (E), ear height (F) and relative chlorophyll content (G) of B104 and *ZmPHR2* overexpression plants. Data are means ± SD (n = 26). (H, I) Morphological changes in chloroplast width (H) and height (I) in BS and MC cells. For BS cells: Chloroplast width and height were measured in B104 (n = 30), *KO-ZmPHR2-1* (n = 27) and *KO-ZmPHR2-2* (n = 27) plants. For MC cells: Chloroplast width and height were analyzed in B104 (n = 25), *KO-ZmPHR2-1* (n = 21) and *KO-ZmPHR2-2* (n = 27) plants. Statistical significance was determined by Students’ ***t***-test. Exact *p* values are directly marked on the (B-I).

**Figure S7. Identification, expression pattern and protein expression levels of *ZmISP* under blue and white light treatments at different time points after RBSDV inoculation.**

(A) Alignment of the peptide sequences of ZmISP and TaISP. Regions highlighted with blue backgrounds indicate highly conserved amino acid residues shared by the two proteins. (B) Schematic diagram of ZmISP domain architecture. Residues 1-46 contain the chloroplast transit peptide (cTP); 54-92 is a CytB6-F_Fe-S domain; 116-198 contain a Rieske domain. (C) RT-qPCR analysis of *ZmISP* in various tissues in B73. Data are shown as means ± SD (n = 3 biological replicates). (D) Immunoblot analysis of ZmISP protein accumulation in maize under RBSDV inoculation (Y) or non-inoculation (N) at 0, 6 and 12 days post inoculation. Actin was used as an internal control, and relative protein levels were normalized to Y 0 d / N 0 d (set to 1.0), quantified using Image J, and shown below the blots. Cropped from original blots with left-right order of the Y and N groups adjusted for presentation. Full-length blots are provided in Source Data. (E) Immunoblot analysis of ZmISP protein accumulation in maize under blue (B 0 h, B 0.25 h, B 0.5 h, B 1 h, B 2 h) and white light (W 0 h, W 0.25 h, W 0.5 h, W 1 h, W 2 h). Cropped from original blots with left-right order of the B and W groups adjusted for presentation. Full-length blots are provided in Source Data. Actin was used as an internal control, and relative protein levels were normalized to B 0 h / W 0 h (set to 1.0), quantified using Image J, and shown below the blots.

**Figure S8. Phenotypic characterization and disease resistance evaluation of *zmisp* mutants.**

(A) Schematic representation of the *ZmISP* gene structure in B73 and *zmisp* mutant. The nucleotide polymorphism responsible for the *zmisp* mutation is indicated by red box. (B, C) Relative expression levels of RBSDV *S6* (B) and *S8* (C) in B73 and *zmisp* mutants at 36 days post-inoculation. Data are shown as means ± SD (n = 3 biological replicates). (D, E) Morphological changes in chloroplast width (D) and height (E) in BS and MC cells. For BS cells: Chloroplast width and height were measured in B73 (n = 30), *zmisp-1* (n = 28) and *zmisp-2* (n = 27) mutants. For MC cells: Chloroplast width and height were analyzed in B73 (n = 29), *zmisp-1* (n = 24) and *zmisp-2* (n = 27) mutants. (F-H) Plant height (F), ear height (G) and relative chlorophyll content (H) of B73 and *zmisp* mutants. Data are means ± SD (n = 23). Statistical significance was determined by Students’ ***t***-test. Exact *p* values are directly marked on the (B-H).

**Figure S9. Phenotypic characterization and disease resistance evaluation of *ZmISP* overexpression plants.**

(A) Western blot analysis showing the expression of *ZmISP* overexpression lines. Anti-GFP antibody was used to detect ZmISP protein and Actin was used as an internal control. (B, C) The relative expression level of RBSDV *S6* (B) and *S8* (C) in B104 and *ZmISP* overexpression lines after RBSDV inoculation. (n = 3 biologically independent samples with six plants per biological replicate). (D-F) Plant height (D), ear height (E) and relative chlorophyll content (F) were analyzed in B104 (n = 22), *OE-ZmISP-1* (n = 23) and *OE-ZmISP-2* (n = 24). Data are means ± SD. Statistical significance was determined by Students’ ***t***-test. Exact *p* values are directly marked on the (B-F).

**Figure S10. *ZmPHR2* and *ZmISP* promotes the production of reactive oxygen species.**

(A, B) Heatmaps display the DEGs related to ROS between B104 and *ZmPHR2* (A), *ZmISP* (B) overexpression plants. Red box indicates up-regulation, blue box indicates down-regulation. (C, D) NBT staining areas of leaves in B104, B73, *ZmPHR2* knockout (C) and *zmisp* mutants (D) after RBSDV inoculation*.* Data are means ± SD, n = 3. (E, F) NBT staining areas of leaves in B104, *ZmPHR2* (E) and *ZmISP* (F) overexpression plants after RBSDV inoculation*.* Data are means ± SD, n = 3. (G) NBT staining of non-inoculated B104, B73, *ZmPHR2* and *ZmISP* overexpression plants, *zmphr2* and *zmisp* mutants*.* Scale bar=3 cm. (H, I) ROS content (H) and NBT staining areas (I) of B104, *ZmPHR2* and *ZmISP* overexpression plants. (J, K) ROS content (J) and NBT staining areas (K) of B73, *zmphr2* and *zmisp* mutants. Data are means ± SD, n = 3. Statistical significance was determined by Students’ ***t***-test. Exact *p* values are directly marked on the (C, D, E, F, H, I, J and K).

**Figure S11. NAC inhibits ROS burst and compromises ZmPHR2 and ZmISP-mediated resistance to RBSDV in maize.**

(A, B) NBT staining and areas of maize leaves after 2 h treatment with different concentrations of NAC (0, 10, 40, 60 mM). Scale bar = 1 cm. (n = 2 biologically independent samples). (C-E) Relative transcript levels of RBSDV *S6* (C), *S8* (D), and *S10* (E) in water- and NAC-treated groups at 12 dpi, as determined by RT‑qPCR. Data are means ± SD, n = 3 biologically independent samples, six plants per biological replicate. Statistical significance was determined by Students’ ***t***-test. Exact *p* values are directly marked on the (B-E).

**Figure S12. RBSDV P6 is an essential effector in RBSDV infection.**

(A) Y2H assay shows the interaction between RBSDV P6 and ZmPHR2. (B) Relative expression level of RBSDV *S6* in B104 and RBSDV P6 overexpression plants after RBSDV inoculation (Data are means ± SD. n = 3 biologically independent samples with six plants per biological replicate). (C-E) Plant height (C), ear height (D) and relative chlorophyll content (E) of B104 (n = 33) and RBSDV P6 overexpression plants (Data are means ± SD. n = 33). (F) NBT staining of B104 and RBSDV P6 overexpression plants without inoculation. Scale bar=3 cm. (G, H) Total ROS content (G) and NBT staining area quantified via ImageJ 1.54 (H) in uninoculated B104 and P6-overexpressing plants (Data are means ± SD. n = 3). Data are means ± SD. Statistical significance was determined by Students’ ***t***-test. Exact *p* values are also directly marked on (B, C, D, E, G and H).

**Figure S13. The subcellular co-localization of ZmPHR2, ZmISP and RBSDV P6.**

GFP fluorescence signals were used to observe the subcellular localization of ZmPHR2 and RBSDV P6 (with FM 4-64 as the membrane marker and chloroplast autofluorescence as the chloroplast marker), and the same signal was also used to observe the subcellular localization of ZmPHR2 and ZmISP (with chloroplast autofluorescence as the chloroplast marker). Scale bar = 10 µm.

**Figure S14. Overexpression o****f *OsISP* or *OsPHR2* enhances rice resistance against RBSDV.**

(A) The plant height of H9508 and RBSDV *P6* overexpression lines (n = 30). Data are means ± SD. (B) The relative expression level of *OsPHR2* in NIP and *OsPHR2* overexpression plants (Data are means ± SD, n = 3 biologically independent samples). (C) The relative expression level of *OsISP* in NIP and *OsISP* overexpression plants. (Data are means ± SD, n = 3 biologically independent samples). (D-E) The relative expression level of RBSDV *S6* (D) and *S8* (E) in NIP, *OsPHR2* and *OsISP* overexpression lines after RBSDV inoculation. (Data are means ± SD, n = 3 biologically independent samples). All data are presented as mean ± SD from three biologically independent samples with six rice seedlings per replicate. Statistical significance was assessed Student’s ***t***-test, and exact *p* values are labeled on each bar graph.

**Supplementary Tables**

**Table S1.** Primers used in this study.

**Table S2.** Sequencing results of positive clones from Y2H library screening.

**Table S3.** Differentially expressed genes in *OE-ZmPHR2* transgenic plants compared to wild-type.

**Table S4.** Differentially expressed genes in *OE-ZmISP* transgenic plants compared to wild-type.

**Table S5.** KEGG pathway enrichment analysis for differentially expressed genes (DEGs) in B104 and *OE-ZmPHR2*.

**Table S6.** KEGG pathway enrichment analysis for differentially expressed genes (DEGs) in B104 and *OE-ZmISP*.
