# Supplementary material for: Blue Light Receptor PHR2‐Mediated Spectrum Antiviral Immunity
Source: Adv Sci (Weinh). 2026 Sep 30:e78050. Online ahead of print. doi: 10.1002/advs.78050 (PMC13626285; doi:10.1002/advs.78050)
Supplement: Supplementary file 2 [file ADVS-9999-e78050-s003.docx]

Figure 2B

①GST+ZmISP-His (Input)

②ZmPHR2-GST+ZmISP-His (Input)

③GST+ZmISP-His (Elution)

④ZmPHR2-GST+ZmISP-His (Elution)

Figure 4C

①GST+P6-His (Input)

②ZmPHR2-GST+P6-His (Input)

③GST+P6-His (Elution)

④ZmPHR2-GST+P6-His (Elution)

Figure 5B

①2×ZmPHR2-GST+ZmISP-His (Input)

②2×ZmPHR2-GST+ZmISP-His+1×P6-His(Input)

③2×ZmPHR2-GST+ZmISP-His+2×P6-His(Input)

④2×ZmPHR2-GST+ZmISP-His+3×P6-His(Input)

⑤2×ZmPHR2-GST+ZmISP-His (Elution)

⑥2×ZmPHR2-GST+ZmISP-His+1×P6-His(Elution)

⑦2×ZmPHR2-GST+ZmISP-His+2×P6-His(Elution)

⑧2×ZmPHR2-GST+ZmISP-His+3×P6-His(Elution)

Figure 6K

①GST+OsISP-His (Input)

②OsPHR2-GST+OsISP-His (Input)

③GST+OsISP-His (Elution)

④OsPHR2-GST+OsISP-His (Elution)

Figure 6L

①GST+P6-His (Input)

②OsPHR2-GST+P6-His (Input)

③GST+P6-His (Elution)

④OsPHR2-GST+P6-His (Elution)

Figure 6M

①2×OsPHR2-GST+OsISP-His (Input)

②2×OsPHR2-GST+OsISP-His+1×P6-His(Input)

③2×OsPHR2-GST+OsISP-His+2×P6-His(Input)

④2×OsPHR2-GST+OsISP-His+3×P6-His(Input)

⑤2×OsPHR2-GST+OsISP-His (Elution)

⑥2×OsPHR2-GST+OsISP-His+1×P6-His(Elution)

⑦2×OsPHR2-GST+OsISP-His+2×P6-His(Elution)

⑧2×OsPHR2-GST+OsISP-His+3×P6-His(Elution)

Figure S4B

Figure S4C

Figure S6A

Figure S7D

Figure S7E

Figure S9A
